# Expanding Immunotherapy Beyond CAR T Cells: Engineering Diverse Immune Cells to Target Solid Tumors

**DOI:** 10.3390/cancers17172917

**Published:** 2025-09-05

**Authors:** Tereza Andreou, Constantina Neophytou, Fotios Mpekris, Triantafyllos Stylianopoulos

**Affiliations:** 1 Cancer Biophysics Laboratory, Department of Mechanical and Manufacturing Engineering, University of Cyprus, Nicosia 2109, Cyprus; 2 Cancer Genetics, Therapeutics & Ultrastructural Pathology Department, The Cyprus Institute of Neurology and Genetics, Nicosia 1683, Cyprus

**Keywords:** chimeric antigen receptor (CAR), cell therapy, immunotherapy, cancer, CAR NK cells, CAR macrophages, CAR dendritic cells

## Abstract

Chimeric antigen receptor (CAR) T cell therapy has revolutionized the treatment of certain hematologic malignancies, yet its success in solid tumors has been limited by antigen heterogeneity, an immunosuppressive tumor microenvironment, and barriers to cell trafficking and persistence. To expand the reach of cellular immunotherapy, multiple immune cell types—γδ T cells, invariant NKT cells, virus-specific T cells, natural killer (ΝΚ) cells, and myeloid effectors such as macrophages and dendritic cells—are now being explored as alternative or complementary CAR platforms. Each lineage brings unique advantages, such as the innate cytotoxicity and safety profile of CAR NK cells, the tissue infiltration and microenvironment-modulating capacity of CAR macrophages, or the MHC-independent recognition offered by γδ T cells. Recent advances in pharmacological strategies, synthetic biology, and artificial intelligence provide additional opportunities to overcome barriers and optimize CAR design and manufacturing scale-up. Here, we review the state of the art in engineering diverse immune cells for solid tumor therapy, highlight safety considerations across autologous, allogeneic, and in vivo CAR cell therapy approaches, and provide our perspective on which platforms might best address current unmet clinical needs. Collectively, these developments lay the foundation for next-generation strategies to achieve durable immunotherapy responses in solid tumors.

## Simple Summary

CAR cell therapy is a cutting-edge treatment that uses genetically modified immune cells to fight cancer. Originally developed using a patient’s own T cells, this approach showed great success in treating blood cancers. Now, scientists are expanding the approach to use other immune cells like natural killer cells, macrophages, and dendritic cells. Each type of cell offers unique benefits. NK cells may be safer, and macrophages can move into solid tumors more easily. Researchers are also exploring ways to use donor cells or even change cells directly inside the body, which could make the treatment faster and easier to use. Despite its promise, CAR therapy still faces challenges like high cost, safety concerns, and how to manufacture it on a large scale. With ongoing research, scientists hope to improve CAR therapies so more patients with hard-to-treat cancers, especially solid tumors (e.g., breast, lung, pancreatic cancers) can benefit in the future.

## 1. Introduction

The immune system is a double-edged sword in cancer development and progression but can also be harnessed for cancer therapy. One of the most significant advances in the field of cancer immunotherapy is the adoptive transfer of immune cells genetically engineered to express chimeric antigen receptors (CARs), which has demonstrated remarkable success, particularly in hematological malignancies [1]. To date, seven CAR T cell products have received FDA regulatory approval in the United States [1]. Despite promising clinical outcomes of CAR T cell therapies in blood cancers, the application of CAR T cell therapies for the treatment of solid tumors faces significant physical, biological, and immunological challenges. Barriers such as the dense tumor stroma and stiff extracellular matrix (ECM) create both physical and immunosuppressive obstacles that hinder the infiltration, persistence, and efficacy of CAR T cells within the tumor microenvironment (TME) [2]. Furthermore, mechanisms of resistance to CAR T cells are increasingly recognized; these include both primary resistance (failure to respond) and acquired resistance (relapse after initial remission) [3]. Several resistance mechanisms have been described, including alterations of the tumor microenvironment, antigen loss or modulation (e.g., downregulation, mutation, or lineage switching), the properties and functionality of the CAR T cells themselves, as well as the function and potential exhaustion of the immune system of the host (extensively reviewed in Andreou et al. 2025 [4]). While numerous strategies are actively being explored to remodel or normalize the TME to potentiate CAR T cell therapeutic efficacy [2], alternative approaches are also gaining attention.

To date, all seven FDA-approved CAR T cell therapies utilize second-generation CAR constructs that incorporate a single co-stimulatory domain (either CD28 or 4-1BB) in combination with the CD3ζ signaling domain, thereby enhancing T cell activation, expansion, and persistence [1]. All currently approved CAR T cell therapies are autologous, meaning they employ the patient’s own T cells to avoid immune incompatibility and minimize the risk of graft-versus-host disease (GvHD) [5]. The approved CAR cell therapy products, to date, are exclusively based on αβ T cells (approximately 90% of the circulating T cells in a healthy individual are of the αβ T cell subtype) and do not include other immune cell subsets, such as other T cell subtypes, natural killer (NK) cells, macrophages or dendritic cells (DCs) nor do they employ allogeneic sources such as donor-derived or induced pluripotent stem cell (iPSC)-derived cells [6,7]. Nevertheless, these alternative platforms are under active investigation in both preclinical models and early-phase clinical trials [8,9,10,11]. In parallel, diverse cellular sources including peripheral blood mononuclear cells (PBMCs), umbilical cord blood, hematopoietic stem cells (HSCs), and iPSCs are actively being explored to optimize the balance between therapeutic efficacy and immune-related toxicities in the clinical setting [12,13,14].

In this review, we highlight emerging CAR-based immunotherapy platforms beyond conventional CAR T cells. We focus on engineered immune cell types including various CAR T cell subtypes, CAR NK cells, CAR macrophages (CAR Ms), and CAR DCs as innovative vehicles for therapeutic payload delivery within solid tumors.

## 2. Autologous CAR Cell Therapy

**Figure 1:**
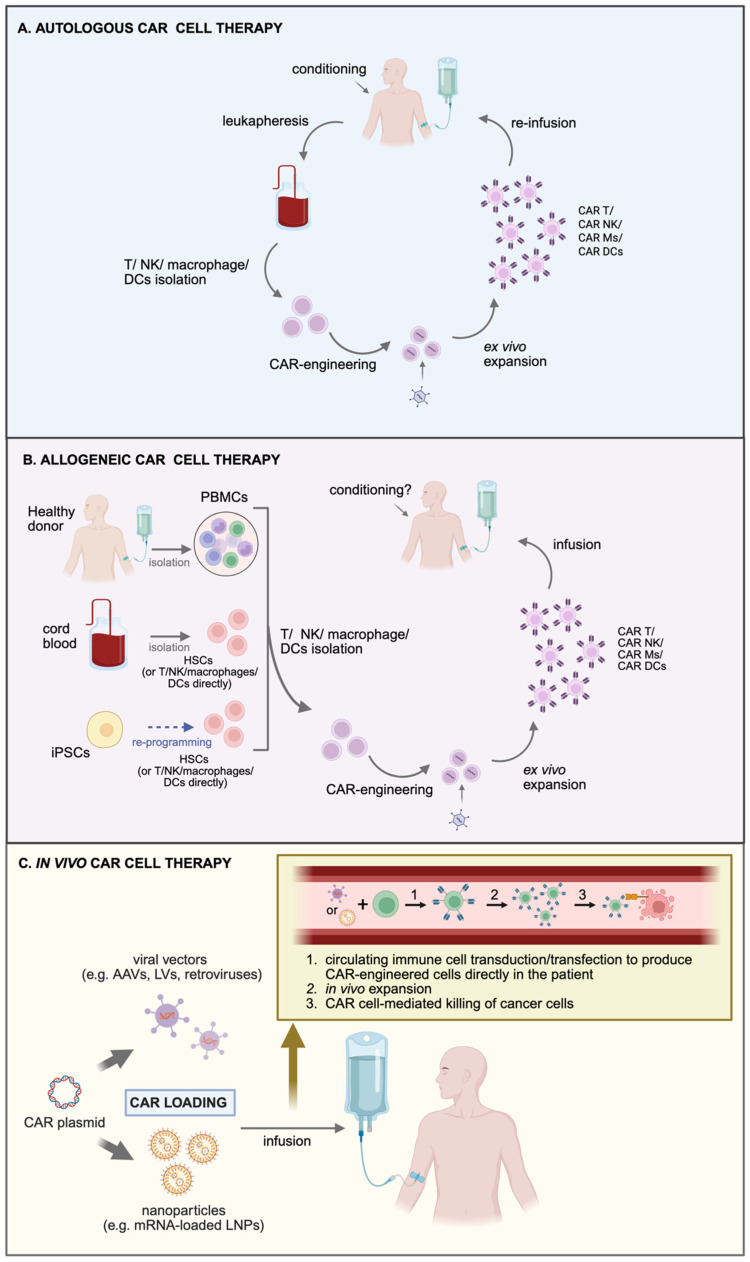
Comparison of autologous, allogeneic, and in vivo CAR cell therapies. (**A**) In autologous CAR cell therapy, the patient’s peripheral blood mononuclear cells are collected by a process called leukapheresis, the desired immune cell population is isolated, modified ex vivo to express CARs, expanded ex vivo, and the CAR-engineered cells are subsequently re-infused into the same patient following a conditioning regimen. (**B**) Allogeneic CAR cell therapy utilizes immune cells from a healthy donor (rather than the patient’s own cells) cord-blood derived immune cells or induced pluripotent stem cells (iPSCs) to generate CAR-engineered cells. (**C**) In vivo CAR cell generation. In vivo CAR cell therapy employs viral vectors or engineered nanoparticles that are administered systemically or locally to deliver CAR constructs directly into immune cells within the patient, thereby bypassing the need for ex vivo modification. These carriers specifically target immune cells to unload their gene editing cargo, leading to the production and subsequent in vivo expansion of CAR engineered cells. The resulting CAR T cells can then specifically detect and kill cancer cells. Abbreviations: AAV, adeno-associated viruses; HSCs, hematopoietic stem cells; iPSCs, induced pluripotent stem cells; LNPs, lipid nanoparticles; LVs, lentiviruses; PBMCs, peripheral blood mononuclear cells. Created in https://BioRender.com (accessed on 28 August 2025).

Autologous CAR cell therapy involves engineering a patient’s own immune cells, typically T cells, to recognize and destroy cancer cells. This personalized approach begins by collecting the patient’s PBMCs by a process called leukapheresis [15]. The desired immune cell population (for example T cells) are then isolated, modified ex vivo to express CARs targeting tumor-associated antigens (TAAs), expanded ex vivo, and the CAR-engineered cells are subsequently re-infused into the same patient following a conditioning regimen (Figure 1A). Typically, the modification methods include viral vectors (such as lentiviruses or retroviruses), mRNA-carrying lipid nanoparticles (LNPs) or using gene editing techniques such as zinc-finger nucleases (ZFNs), and TALENs- and CRISPR/Cas-based platforms [16,17,18,19].

A key advantage of autologous CAR cell therapy is the inherent immunologic compatibility of the CAR-engineered cells with the host, as they are derived from the patient’s own immune system [20]. Since the modified cells originate from the patient, they are inherently compatible with the host immune system, minimizing the risk of GvHD. This has made autologous CAR T therapies like tisagenlecleucel (marketed as Kymriah) and axicabtagene ciloleucel (marketed as Yescarta) highly successful in treating relapsed or refractory B cell leukemias and lymphomas.

However, autologous CAR cell therapy also presents significant challenges. The manufacturing process is complex, costly, and time-consuming (often taking several weeks), which can be critical for patients with aggressive disease [21]. Moreover, individual patient characteristics and pretreatments can directly impact the efficacy and quality of the autologous CAR cell product [22]. Chemotherapy, especially aggressive regimens or depletion approaches, can negatively impact immune cell quality, which poses a challenge in CAR engineering [22]. Cytotoxic agents can induce apoptosis and contribute to sustained lymphopenia, thereby reducing the availability of healthy immune cells for CAR engineering [23]. In addition to direct cytotoxicity, chemotherapy can impair immune cell trafficking, disrupt the balance of immune cell subsets, and dampen the overall immune response [24,25,26]. Furthermore, chemotherapy can alter the TME; for example, by increasing immunosuppressive cell populations (such as regulatory T cells (Tregs), myeloid-derived suppressor cells, and tumor-associated macrophages), or via stromal remodeling [27,28,29]. The aforementioned changes may potentially hinder the expansion, persistence, and antitumor activity of CAR-engineered cells.

Another concern is immune-related toxicities which can be life-threatening. The most notable and most studied immune-related adverse effects related to autologous CAR-engineered cells are cytokine release syndrome (CRS) and immune effector cell-associated neurotoxicity syndrome (ICANS), which require intensive monitoring and management [30]. CRS is an acute systemic inflammatory response characterized by fever, systemic inflammation, and in severe cases, multiple organ dysfunction. ICANS is characterized by a constellation of neuropsychiatric symptoms, ranging from mild to severe, and affects approximately 20–70% of CAR T cell therapy recipients [31]. Finally, logistics such as individualized production and transportation, further complicate the widespread clinical application of autologous CAR cell therapies [32].

Despite these limitations, autologous CAR cell therapy remains a groundbreaking advancement in cancer treatment, particularly for hematologic malignancies. Ongoing innovations aim to improve efficacy, reduce manufacturing time, and expand applicability to solid tumors [33,34]. Alternatives such as allogeneic (“off-the-shelf”) CAR therapies, and alternative host cells, such as CAR-engineered γδ T cells, CAR NK cells, CAR Ms, and more recently, CAR DCs, are under investigation to overcome current barriers while retaining therapeutic benefits.

## 3. Allogeneic CAR Cell Therapy: Promise and Challenges

Contrary to autologous CAR cell therapy, allogeneic CAR cell therapy utilizes immune cells from a healthy donor (rather than the patient’s own cells) to generate engineered cells capable of targeting cancer cells [35]. This allogeneic, or “off-the-shelf” approach, contrasts with autologous CAR cell therapies, offering the potential for immediate availability, standardized manufacturing, and reduced costs—key advantages for patients with aggressive or rapidly progressing disease, for whom timely treatment initiation is critical.

The manufacturing process for allogeneic CAR cell products involves the isolation of T cells or alternative immune cell populations (such as γδ T cells, NK cells, or HSCs) from healthy donors (Figure 1B). These cells are subsequently engineered to express CARs specific to TAAs, enabling targeted cytotoxicity against malignant cells. To mitigate the immunologic risks associated with allogeneic transplantation, including host-versus-graft (HvG) immune rejection and GvHD, additional genetic modifications are commonly incorporated. These may involve targeted disruption of endogenous T cell receptor (TCR) α/β chains via gene editing technologies such as CRISPR/Cas9, TALENs, or ZFNs, thereby preventing alloreactivity [35]. Alternatively, the use of inherently non-alloreactive cell types (e.g., γδ T cells, NK cells, macrophages) can eliminate the need for TCR modification and can make allogeneic CAR cell products safer and more universally applicable [36]. Collectively, these strategies aim to generate “off-the-shelf” CAR cell therapies that are both efficacious and safe for administration across HLA-mismatched recipients.

One of the most promising aspects of allogeneic CAR cell therapies is the potential for large-scale, centralized manufacturing, enabling the generation of standardized, cryopreservable cell banks suitable for widespread clinical deployment [37]. This “off-the-shelf” capability facilitates immediate treatment access, bypassing the individualized collection and production timelines required for autologous therapies. This also enables treatment of patients who are heavily immunocompromised, lymphopenic, or have insufficient T cell quality for autologous therapy [38]. Early-phase clinical trials of allogeneic CAR T and CAR NK therapies have shown encouraging safety profiles and antitumor activity [39,40]. As technologies advance, allogeneic CAR-engineered cells hold promise as a scalable, readily available, and broadly accessible immunotherapy platform capable of expanding the reach of cell therapies to a wider range of cancers and patient populations.

## 4. In Vivo CAR Gene Therapy: Potential and Challenges

Traditional CAR T cell therapies involve ex vivo modification and expansion of autologous T cells, a process that is both time-consuming and resource-intensive. Recently, in vivo CAR gene therapy has emerged as a novel approach, in which vectors are administered systemically or locally to directly reprogram immune cells within the patient, thereby bypassing the need for ex vivo manipulation (Figure 1C) [41]. This strategy holds considerable promise for the treatment of hematological malignancies and autoimmune diseases, as it offers the potential for scalable, “off-the-shelf” CAR therapies and could facilitate localized immune responses by generating CAR-modified T or NK cells in situ [42,43]. Advances in gene delivery platforms such as adeno-associated viruses (AAVs), lentiviral vectors, LNPs, and synthetic polymers have enabled targeted transduction of specific immune cell populations, paving the way for direct in vivo CAR engineering [44,45,46,47]. One such investigational therapy is INT2104, a lentiviral-based product, which is administered as a single intravenous dose to patients and works by creating CAR T cells and CAR NK cells in the patient’s body, which set out to target CD7, a protein that is highly expressed in blood cancers (INVISE study, NCT06539338). Preclinical studies have shown that a single dose of INT2104 was able to destroy B cells and get rid of tumors in mouse models, and a toxicology study demonstrated INT2104′s safety profile in nonhuman primates. Importantly, in vivo CAR therapies can be administered to patients without lymphodepletion beforehand (as this would deplete the very cells that need to be modified) [40]. The patients maintain an intact immune system and therefore are less prone to CRS and ICANS as healthier immune cells may put a brake to excessive release of cytokines. On the downside, a fully functional immune system may reject either the vector or the CAR it encodes, rendering such in vivo approaches ineffective.

However, applying in vivo CAR cell therapy to solid tumors (for instance in breast cancer, lung cancer, or pancreatic cancer) introduces distinct challenges. One of the main challenges is the highly immunosuppressive TME, which impairs immune cell infiltration, persistence, and function [48]. Moreover, factors such as hypoxia, immunosuppressive cytokines, regulatory immune cells, and the dense/stiff ECM collectively hinder CAR cell activity following in vivo delivery [41,49]. In addition, physical barriers such as the abnormal tumor vasculature further restrict the trafficking of CAR-engineered cells to tumor sites [50].

A wide range of pharmacological strategies are being actively explored to overcome the immunosuppressive microenvironment of solid tumors and thereby enhance the efficacy of CAR-engineered immune cells (extensively reviewed in Andreou et al., 2025 [4]). Immune checkpoint blockade with antibodies targeting PD-1/PD-L1 or CTLA-4 can reinvigorate exhausted T cells [51]. TGF-β pathway inhibition, using small molecule inhibitors can counteract TGF-β-mediated exclusion and dysfunction of lymphocytes [52]. Vascular normalization strategies, using agents such as bevacizumab (an anti-VEGF monoclonal antibody), combretastatin A-4 phosphate (a vascular disrupting agent), and NEO100 (a blood–brain barrier permeabilizer), aim to improve perfusion, reduce VEGF-driven immunosuppression, and enhance T cell extravasation and trafficking into tumors [53,54,55]. Furthermore, agents that target the ECM, such as hyaluronidase, aim to decompress the dense tumor stroma and improve immune cell penetration [56]. Innate immune stimulation through STING agonists or TLR9 agonists can convert “cold” tumors into “hot” tumors, thereby enhancing antigen presentation and synergizing with immune checkpoint inhibitors [57,58]. Finally, reprogramming of immunosuppressive myeloid cells (e.g., CSF1R or PI3K-γ inhibitors) can deplete or repolarize tumor-associated macrophages, while blockade of chemokine receptors such as CXCR1/2 or CCR2 reduces the recruitment of myeloid-derived suppressor cells [59,60,61]. Together, these pharmacological approaches exemplify rational methods to remodel the TME and provide opportunities to augment CAR-based therapies in solid cancers.

Another critical challenge is the antigenic heterogeneity commonly observed in solid tumors. In certain hematologic malignancies, target antigens such as CD19 are often expressed at high and relatively homogeneous levels at diagnosis, enabling effective targeting by CAR T cells; however, antigen loss and clonal diversity may still occur. In contrast, solid tumors typically display greater spatial and temporal heterogeneity in tumor-associated antigen (TAA) expression, contributing to incomplete tumor targeting and immune escape [62,63,64,65,66,67]. This heterogeneity compromises the effectiveness of CAR T cells generated in vivo, as insufficient recognition of diverse tumor cell populations can result in suboptimal tumor clearance and disease relapse and can increase the risk of on-target, off-tumor toxicity. To address this issue, advanced CAR engineering strategies are under investigation. These include the co-expression of multiple CARs within a single T cell, as well as the development of tandem or bispecific CARs that incorporate multiple antigenrecognition domains [68]. Such modifications aim to broaden the target spectrum, enhance tumor cell recognition despite antigen heterogeneity, and reduce the likelihood of immune evasion. Precision in vector targeting is essential to ensure selective transduction of circulating T cells without affecting other cell types, which remains a technical hurdle. In addition, when using lentiviral vector-based approaches, vector integration into the immune cell genome may be a cancerous event (in rare cases) compromising the safety of this approach [69]. Furthermore, regulating CAR expression in vivo, for example, by expressing the payload under specific promoters or by incorporating ON/OFF switches in CAR designs, is critical to enhance safety and efficacy, especially given the variable and often less durable responses observed in solid tumors [70,71]. Looking forward, successful translation of in vivo CAR gene therapies to solid tumors will require innovations in vector engineering, tumor antigen targeting, and synthetic gene circuit design to allow for precise and targeted CAR expression.

## 5. Beyond Conventional CAR T Cells: Alternative T Cell Platforms for CAR Engineering

**Figure 2:**
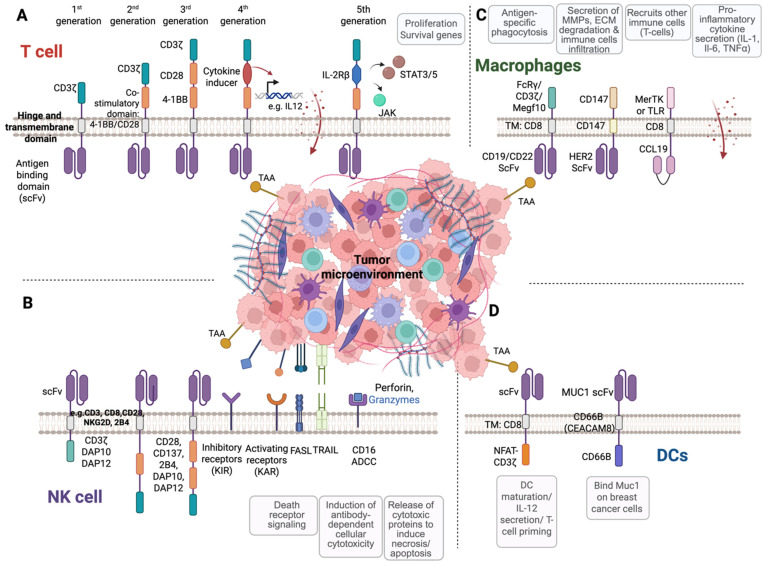
The evolution of CAR-engineered cells. Structure and evolution of chimeric antigen receptors (CARs) across immune cell types. (**A**) Evolution of CAR designs in T cells. CARs typically consist of an extracellular antigen-binding domain, commonly a single-chain variable fragment (scFv) or a functional ligand domain, a transmembrane hinge region, and an intracellular signaling domain. The intracellular domain has evolved over five generations. (**B**) Structure and function of CAR NK cells. The design of CAR NK cells was initially adapted from CAR T cell architecture, comprising an extracellular antigen-binding domain, commonly scFv, for tumor antigen recognition, a transmembrane domain derived from receptors such as NKG2D, CD3, CD8, CD28, or 2B4, and an intracellular signaling region. (**C**) Design and functional diversity of CAR constructs in macrophages. CAR macrophages (CAR Ms) are composed of an extracellular antigen-binding domain, a hinge region, a transmembrane domain, and a cytoplasmic signaling domain. (**D**) Structure of CAR-modified dendritic cells (CAR DCs). CAR DCs are engineered with an extracellular scFv targeting tumor-associated antigens (such as MUC1), a transmembrane domain commonly derived from CD8α, CD28, or CD66b, and an intracellular signaling domain. Created in https://BioRender.com (accessed on 28 August 2025).

**Figure 3:**
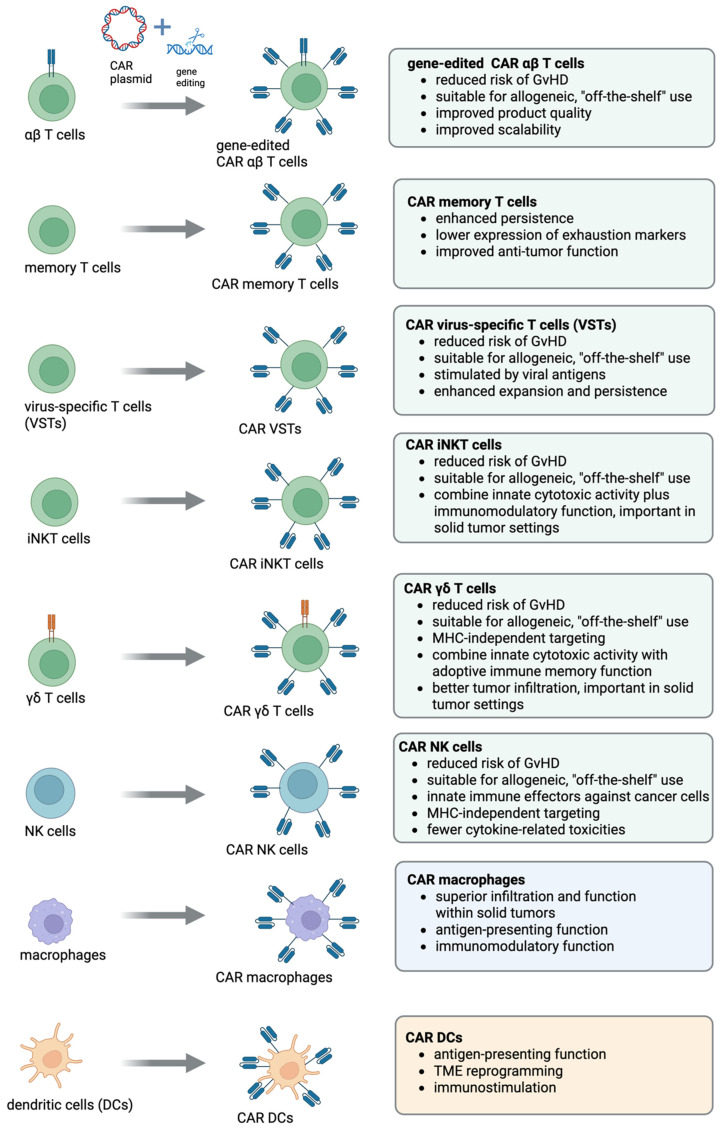
Alternative CAR cell platforms. An overview of alternative CAR cell platforms, outlining their main advantages over conventional CAR αβ T cells. Created in https://BioRender.com (accessed on 28 August 2025).

CAR T cell therapy has revolutionized the treatment landscape for certain hematologic malignancies, primarily through the use of genetically modified αβ T cells and second-generation CAR vector designs. However, the limitations associated with CAR αβ T cells such as major histocompatibility complex (MHC) restriction, risk of GvHD, and susceptibility to the immunosuppressive TME have spurred interest in alternative host cell types. Beyond conventional αβ T cells and the development of progressively advanced vector designs (third-, fourth-, and fifth- generation CAR T vectors) (Figure 2), alternative T cell subtypes such as gene-edited αβ T cells, memory T cells, virus-specific T cells (VSTs), invariant natural killer T (iNKT) cells, and γδ T cells are being actively investigated as platforms for CAR engineering to address limitations in persistence, safety, and efficacy associated with first-generation CAR T cell therapies (Table 1 and Figure 3).

### 5.1. Gene-Edited αβ T Cells

Gene editing of T cells to reduce the risks of GvHD and host immune rejection has emerged as one of the most promising and widespread strategy for the development of universal, “off-the-shelf” CAR T cell products. Given that GvHD is largely driven by TCR recognition of host tissue, gene editing approaches focused on disrupting the endogenous TCR components [83]. Multiple studies have explored disrupting the TCR constant alpha chain (TRAC) or beta chain (TRBC). Torikai et al. showed that ZFN-mediated knockout of the αβ TCR from CD19-CAR T cells did not significantly alter the cells’ ability to kill CD19-positive targets [84]. More recently, with advancement in gene editing techniques, various studies have demonstrated knockout of TRAC using TALENs and CRISPR/Cas9 approaches [85,86]. In addition to gene editing strategies, protein engineering approaches have been developed to retain the TCR within the endoplasmic reticulum rather than being exposed on the surface of T cells, using an anti-TCR linked to the KDEL motif [35]. While these techniques have become increasingly efficient, any remaining T cells that continue to express ab TCR can be removed by magnetic cell separation ex vivo using anti-abTCR antibodies. Stenger et al. showed that TCR-knockout CAR T cells retained potent antileukemic activity while minimizing alloreactivity [87]. However, these gene-edited cells demonstrated reduced persistence in vivo compared to endogenous TCRs, raising concerns about this method. Furthermore, modification to the endogenous TCR does not address the issue of immunogenicity. To decrease immunogenicity, researchers have targeted b-2 microglobulin (B2M), a component of HLA class I molecules that is present on all T cells [88]. Ren et al. showed that CAR T cells including knockout of B2M had reduced alloreactivity in vivo [89]. Further refinements include dual knockout strategies, such as the combined deletion of B2M and the class II transactivator (CIITA), which eliminates both HLA class I and class II expression, which results in improved persistence and reduced immunogenicity in vitro [90].

### 5.2. Memory T Cells

Memory T cells, particularly central memory (T_CM_) and stem cell memory (T_SCM_) subsets, offer another promising source for CAR engineering. These cells are characterized by increased proliferation potential, persistence, and resistance to exhaustion, which are critical attributes for long-term tumor control, especially in solid tumor settings [72]. CAR T cells derived from enriched memory populations have shown superior in vivo expansion and durability compared to bulk T cell products, which often include terminally differentiated or senescent T cells with limited functional capacity [73]. Moreover, memory-derived CAR T cells may be more resilient to the hostile TME, providing sustained antitumor responses with a potentially improved safety profile [74]. Several studies have highlighted that generating CAR T cells from T_CM_ populations (CD45RO+/CD62L+ or CCR7+) or T_SCM_ populations is associated with improved CAR T cell effector function [91,92,93]. Other studies have shown that CD45RA-negative T cells expressing either a NKG2DL-specific or CD19-CAR have anti-cancer effects and decreased in vivo and in vitro alloreactivity [94,95,96]. Using a similar approach, CD19-CAR-engineered CD27-negative T cells (effector and terminal effector memory subsets) have also shown promise in preclinical models [35].

### 5.3. Virus-Specific T Cells (VSTs)

Virus-specific T cells (VSTs) have demonstrated a favorable safety profile and clinical efficacy in diverse patient populations. Across multiple clinical studies, the incidence of GvHD associated with allogeneic VST infusion has remained remarkably low [75]. This success has led to the establishment of VST banks, facilitating the availability of “off-the-shelf” allogeneic VST products. Although the precise mechanisms underlying this safety are not fully elucidated, it is hypothesized that the limited TCR diversity characteristic of memory VSTs reduces their alloreactive potential.

Building on the safety and feasibility of VSTs, interest has emerged in using them as platforms for CAR engineering. Autologous CAR-transduced VSTs targeting TAAs such as CD30 (Hodgkin lymphoma), HER2 (glioblastoma), and GD2 (neuroblastoma and osteosarcoma) have been successfully manufactured and infused in early-phase clinical studies, demonstrating favorable safety and preliminary signs of efficacy [97,98,99]. In post- hematopoietic cell transplantation (HCT) patients, donor-derived VSTs have also been used to generate CD19-targeted CAR VSTs. In one example, PBMCs collected from the original HCT donor were used to manufacture CD19 CAR VSTs [76]. Manufacturing time for this product was significant, requiring culture for 5–6 weeks. Upon infusion into patients with B cell malignancies, the therapy was well-tolerated, with no observed GvHD and evidence of both anti-leukemic activity and retained viral specificity. Further support for this platform comes from an ongoing clinical trial (NCT01430390) assessing allogeneic Epstein–Barr virus (EBV)-specific T cells engineered with a CD19 CAR [35]. In this study, VSTs were derived either from the original HCT donor or from partially matched third-party donors when the original donor was unavailable. Both donor types yielded encouraging clinical responses with minimal toxicity. Beyond EBV, clinical-grade VSTs have been most commonly generated against CMV (pp65/IE1) and adenovirus (hexon/penton), and incorporated into multivirus products that also recognize BK polyomavirus and HHV-6; these have demonstrated safety and high response rates after HSCT, including as banked third-party products [100,101]. In virus-associated cancers, EBV-specific CTLs can directly lyse tumor cells and produce durable remissions. For non-viral tumors, CAR-modified VSTs couple antiviral TCRs with tumor-directed CARs. Early clinical data with HER2-CAR VSTs in glioblastoma show feasibility, safety, and evidence of clinical benefit, and CMV-specific CAR-T platforms are being explored for vaccine-mediated in vivo boosting [99,102,103]. Preliminary clinical data indicate that allogeneic CAR-transduced VSTs can mediate potent antitumor activity while maintaining a low risk of GvHD. The low incidence of GvHD with VSTs likely reflects enrichment for antigen-experienced, virus-restricted TCRs with limited alloreactivity. In a Phase II study of third-party, pentavalent VSTs, only two cases of de novo grade-1 GvHD were observed among 38 treated patients, despite partial HLA matching, with functional persistence for up to 12 weeks [104]. A unique advantage of this approach is the retention of viral specificity, which can provide periodic antigenic stimulation in vivo, thereby supporting CAR T cell expansion and persistence [105]. Nonetheless, current data predominantly involve donor-derived products, which likely mitigate immunologic barriers such as rejection and alloimmunization.

### 5.4. Invariant Natural Killer T (iNKT) Cells

iNKT cells are a rare T cell subset with features of both NK cells and T cells. iNKTs are restricted by CD1d, a glycolipid presenting HLA I-like molecules expressed on B cells, antigen-presenting cells, and some epithelial tissues [106]. Since iNKT express an invariant TCR, they do not cause GvHD and have been shown to confer GvHD protection in allogeneic HCT settings. iNKT cells have been shown to be decreased in number and defective in cancer patients [107]. Preclinical studies have demonstrated the antitumor efficacy of CAR-engineered iNKT cells targeting CD19 and GD2 in murine models of lymphoma and neuroblastoma, respectively [77,108]. Their lack of GvHD induction makes them an attractive platform for allogeneic “off-the-shelf” CAR therapies [109]. Preliminary results from a clinical trial evaluating autologous GD2-CAR iNKT cells co-expressing IL-15 (NCT03294954) in pediatric neuroblastoma have demonstrated safety and feasibility of this approach [110]. While clinical data on allogeneic CAR iNKT cells remain pending, an ongoing trial evaluating allogeneic CAR19-iNKT cells for hematologic malignancies (NCT03774654) is expected to provide insight into their safety and therapeutic potential.

### 5.5. γδ T Cells

CAR-engineered γδ T cells have emerged as a promising platform, offering several distinct advantages that could enhance the safety, efficacy, and applicability of CAR T therapies [111]. γδ T cells represent a distinct T cell lineage with innate-like cytotoxicity and HLA-independent tumor recognition. One of the most compelling features of γδ T cells is their major histocompatibility complex (MHC)-independent antigen recognition [112]. Unlike αβ T cells, γδ T cells do not require antigen presentation via classical MHC molecules, reducing the risk of alloreactivity and making them suitable for “off-the-shelf” allogeneic applications [113,114]. This property allows CAR γδ T cells to target tumor cells that evade immune detection by downregulating MHC expression, a common resistance mechanism in both solid and hematologic malignancies. Moreover, γδ T cells exhibit low alloreactivity, significantly reducing the risk of GvHD in allogeneic settings [115]. This feature makes CAR γδ T cells particularly well-suited for the development of universal, “off-the-shelf” therapies, circumventing the logistical and manufacturing challenges associated with autologous CAR αβ T cell products [114]. In addition to their MHC-independence and reduced GvHD potential, γδ T cells possess intrinsic antitumor activity through their innate-like recognition of transformed cells [78,116,117,118]. This endows CAR γδ T cells with a dual mechanism of action: one mediated through the CAR and the other via their native TCR and associated receptors. Such redundancy could enhance therapeutic efficacy, especially in heterogeneous TMEs where CAR-targeted antigen expression may be variable.

Another advantage lies in the broader tumor recognition profile of certain γδ T cell subsets, particularly Vγ9Vδ2 cells, which are capable of responding to a variety of tumor types due to their sensitivity to metabolic dysregulation and stress ligands [119,120]. This broad reactivity may allow CAR γδ T cells to be applied across a wider spectrum of malignancies. Importantly, γδ T cells may demonstrate greater resistance to the immunosuppressive TME, including factors such as transforming growth factor beta (TGF-β), hypoxia, and adenosine, which often impair αβ T cell function. Preliminary studies suggest that γδ T cells maintain their cytotoxic activity and proliferative capacity under conditions of hypoxia, contributing to more durable antitumor responses [121]. However, the cytokine profile and cytotoxicity of γδ T cells are seemingly determined by cross-talk with microenvironment components [122]. From a manufacturing perspective, γδ T cells typically exhibit faster in vitro expansion kinetics compared to αβ T cells [123], which could streamline production and reduce time to treatment, a critical factor in rapidly progressing cancers. Finally, the safety profile of CAR γδ T cells may be improved relative to CAR αβT cells, particularly with regard to the incidence and severity of CRS and ICANS. Although clinical data remain limited, early-phase trials and preclinical studies suggest that γδ T cells may elicit a more controlled cytokine response.

Preclinical studies have demonstrated that CAR-modified Vγ9Vδ2 T cells retain their intrinsic tumor-recognition capacity while gaining enhanced specificity through CAR redirection [124,125]. For example, CAR γδ T cells targeting GD2, CD19, and NKG2D ligands have shown potent antitumor activity in xenograft models of neuroblastoma, leukemia, and solid tumors, often with reduced risk of graft-versus-host disease compared with αβ T cells [126,127,128]. Early clinical translation is underway: a first-in-human Phase I trial of GD2-targeted CAR γδ T cells (NCT04165941) in relapsed/refractory neuroblastoma is ongoing, with preliminary reports indicating feasibility and manageable safety. Additional trials are testing CD19-directed CAR γδ T cells in B cell malignancies (NCT04735471) and exploring allogeneic, off-the-shelf CAR γδ products derived from healthy donors. In summary, CAR γδ T cells offer several theoretical and practical advantages over traditional CAR αβ T cells, including MHC-independent targeting, lower GvHD risk, innate tumor recognition, broad-spectrum antitumor activity, improved resistance to immunosuppression, faster manufacturing, and potentially reduced toxicity.

Collectively, the aforementioned alternative T cell platforms offer multiple advantages over conventional CAR αβ T cells, including enhanced persistence, reduced risk of GvHD, broader tumor recognition, and potential for universal, “off-the-shelf” therapy. Ongoing clinical studies are expected to elucidate the safety, feasibility, and comparative efficacy of these next-generation CAR-engineered cell products.

## 6. CAR NK Cells: A Promising Alternative to Address Limitations of CAR T Cell Therapy

As an alternative to CAR T cells, CAR NK cells are emerging as a promising immunotherapeutic platform (Table 1 and Figure 3). Unlike T cells, NK cells can recognize and eliminate abnormal cells without the need for antigen presentation via the MHC, significantly reducing the risk of GvHD [129]. This enables the use of allogeneic sources such as immortalized NK cell lines (such as NK-92) [130,131], umbilical cord blood [132,133], iPSCs [134], and human embryonic stem cells (hESCs), supporting scalable “off-the-shelf” manufacturing [129]. In contrast to the complex and individualized production process of CAR T cells, which often faces delays due to low peripheral T cell counts in heavily pre-treated patients, CAR NK cells offer a more accessible and efficient alternative [135]. Importantly, CAR NK cells also display a favorable safety profile, with reduced risk of CRS and ICANS, attributed to their distinct cytokine secretion and shorter in vivo persistence [129].

### 6.1. Structural Design and Generational Advances of CAR NK Cells

Most current CAR NK constructs are adapted from CAR T cell designs, but these are not fully optimized for NK cell biology. Given the distinct repertoire of activating receptors and adapter proteins of NK cells, tailored CAR designs may significantly enhance their therapeutic potential [136]. In particular, fine-tuning extracellular, transmembrane, and intracellular domains could improve activation and persistence of CAR NK cells (Figure 2B).

Early constructs typically used CD3ζ alone, but newer generations incorporate NK-specific signaling molecules such as DAP10 or DAP12, which improve cytotoxicity and persistence [82,137]. Costimulatory domains such as 4-1BB, CD28, OX40, ICOS, or MYD88-CD40 have also been explored. Parallel to CAR T evolution, first-generation CAR NKs used only CD3ζ, second-generation CAR NKs added one costimulatory domain, while third-generation constructs combine multiple signals. Armored CAR NKs encoding IL-15 have shown remarkable in vivo persistence, with some patients maintaining NK cells for over a year, accompanied by robust antitumor activity but without CRS, neurotoxicity, or GvHD [39,138]. In addition, cytokine-induced memory-like NK cells, generated via IL-12/15/18 priming, display prolonged persistence and enhanced cytotoxicity [139,140].

While most CAR NK constructs employ single-chain variable fragments (scFvs) as the extracellular antigen recognition domain, alternative designs are being developed to optimize specificity, stability, and reduce immunogenicity. Nanobodies derived from camelid heavy-chain antibodies have been used to generate more compact CARs with improved tumor penetration [141]. Ligand-based recognition domains are another strategy; for example, NKG2D-CARs exploit NK cell-specific pathways to detect stress-induced ligands such as MICA/B and ULBPs, showing efficacy in preclinical solid tumor models [142,143,144]. Similarly, IL-13 muteins have been engineered into CARs for selective targeting of IL13Rα2-expressing gliomas, while natural receptor ectodomains such as NKp30 or NKp44 have been incorporated to leverage endogenous NK recognition mechanisms [145,146,147]. These alternative extracellular domains broaden the landscape of targetable antigens and may enhance the adaptability of CAR NK platforms across diverse malignancies. However, careful consideration of safety is essential, as some stress-induced ligands recognized by NKG2D or NKp30 are also expressed on healthy tissues under inflammatory conditions, raising the potential for on-target, off-tumor toxicity. Thus, while these strategies expand therapeutic possibilities, their clinical translation requires rigorous preclinial evaluation and rational engineering to minimize unwanted side effects.

These advances highlight the value of tailoring extracellular, transmembrane, and intracellular CAR domains specifically for NK cells rather than relying on T cell-derived templates.

### 6.2. Functional Advantages and Challenges of CAR NK Cells

CAR NK cells possess several functional properties that distinguish them from conventional CAR T cells and contribute to their growing appeal as a therapeutic platform. Beyond CAR-directed cytotoxicity, NK cells retain their innate capacity to recognize malignant cells through activating receptors such as NKG2D, DNAM-1, and killer immunoglobulin-like receptors, as well as through antibody-dependent cellular cytotoxicity [148,149,150,151]. This dual mechanism provides a degree of redundancy that could prove advantageous in heterogeneous tumors where CAR-targeted antigens may be variably expressed. Compared to CAR T cells, CAR NK cells generally exhibit a more favorable safety profile, with markedly lower incidence of CRS and ICANS [129,135,152]. This is attributed to their distinct cytokine secretion pattern and relatively shorter in vivo persistence, which limits long-term off-target effects. Importantly, their reduced risk of GvHD disease enables the use of allogeneic sources, supporting the development of scalable “off-the-shelf” products.

Despite these strengths, several barriers remain to the widespread clinical success of CAR NK cells. Their trafficking and infiltration into solid tumors is frequently suboptimal, although this can be improved by engineering expression of chemokine receptors such as CXCR4, CXCR1, or CXCR2 to match tumor-secreted ligands [79,80,81,153,154]. Transduction efficiency also lags behind that of T cells, but has been enhanced by using baboon envelope-pseudotyped lentiviral vectors, transduction enhancers like retronectin and ectofusin-1, or non-viral systems including Sleeping Beauty transposons [135]. In addition, the immunosuppressive TME impairs NK activity through factors such as TGF-β, adenosine, and hypoxia [155,156,157]. To counteract this, strategies under investigation include blockade of TGF-β signaling, engineering resistance to adenosine, and combining CAR NK therapy with checkpoint inhibitors targeting PD-1, CTLA-4, NKG2A, TIGIT, and TIM-3 [158,159,160,161]. While CAR macrophages share some of these challenges, CAR NK cells are capable of more rapid cytotoxicity and are more amenable to universal allogeneic application, though they lack the phagocytic and antigen-presenting functions that macrophages can provide.

### 6.3. Recent Advances in NK Cells’ Antitumor Activity

Preclinical work has shown potent CAR NK activity in hematological and solid tumors. Clinical validation came from the CD19/IL-15 CAR NK trial using cord blood-derived NKs (NCT03056339), which reported durable responses without severe toxicities [39]. Additional early-phase trials are testing CAR NKs against glioblastoma, ovarian, and hepatocellular carcinoma, with encouraging safety data. Novel approaches include NK cells engineered with resistance to TGF-β or checkpoint inhibition, and iPSC-derived CAR NKs that allow standardized, large-scale production [82]. Furthermore, NK-recruiting antibodies that release chemokines (e.g., CXCL16) in the TME are being developed to enhance trafficking [162].

Taken together, these advances are helping to overcome the current limitations of CAR NK cells and position them as a safer, scalable, and potentially more effective alternative to CAR T cells, particularly in the context of solid tumors, where CAR T therapies have faced limited success. Building on the success of NK cell engineering, recent efforts have also focused on macrophages, whose intrinsic tumor-homing, phagocytic, and microenvironment remodeling capacity provide a distinct but complementary avenue for CAR-based immunotherapy.

## 7. Engineering CAR Macrophages (CAR Ms) for Enhanced Antitumor Immunity

CAR Ms hold significant promise for the treatment of solid tumors, leveraging the innate tumor-homing ability and functional versatility of macrophages (Table 2 and Figure 3). Macrophages are often the most abundant immune cells within the TME [163], where they contribute to ECM remodeling and facilitate immune cell infiltration. Macrophages play a dual role in the TME depending on their polarization state. Classically activated M1-like macrophages exhibit pro-inflammatory and antitumor activity, while alternatively activated M2-like macrophages (also known as tumor-associated macrophages; TAMs) support tumor progression through immunosuppression, angiogenesis, and TME remodeling [164]. Reprogramming macrophages and shifting the balance towards the M1-like phenotype represents a promising therapeutic strategy to overcome tumor-induced immunosuppression [165]. By enhancing pro-inflammatory signaling and antigen presentation, M1-polarized macrophages can reinvigorate antitumor immunity and improve the efficacy of immunotherapies within the TME. Consequently, CAR Ms are emerging as a novel and complementary therapeutic platform given their natural tumor-homing properties, ability to remodel the TME, and capacity for sustained antigen presentation. Insertion of a CAR on macrophages could allow them to selectively recognize and phagocytose antigen overexpressing cancer cells.

### 7.1. Structural Design and Generational Advances of CAR Ms

CAR Ms share a similar structural framework with CAR T cells, consisting of an extracellular antigen-binding domain, a hinge region, a transmembrane domain, and an intracellular signaling domain (Figure 2C). However, the key distinction lies in the adaptation of intracellular signaling components to suit macrophage-specific biology. While the CD3ζ chain, commonly used in CAR T cells, can also be incorporated into CAR Ms, it engages a different downstream pathway in macrophages. Instead of the ZAP-70 kinase found in T cells, CD3ζ signals through the Syk tyrosine kinase in macrophages to initiate phagocytic activity [173]. In addition to CD3ζ, other intracellular domains such as the Fc receptor γ chain (FcRγ) and multiple epidermal growth factor-like domains protein 10 (Megf10) have been effectively utilized in CAR M constructs [166,174]. These signaling motifs activate native macrophage mechanisms, promoting robust phagocytosis of tumor cells upon antigen engagement [175]. Incorporation of co-stimulatory signaling domains, similar to those found in second- and third-generation CAR T cells (Figure 2), further amplifies macrophage functionality. For example, the fusion of a phosphoinositide 3-kinase (PI3K)-recruiting motif to a CAR-FcRγ construct has been shown to significantly enhance the phagocytosis of intact tumor cells [176], underscoring the potential of rational CAR design tailored to macrophage biology.

Building on this foundational design, CAR Ms have progressed through three generations to improve their antitumor efficacy. First-generation CAR Ms focused on enabling phagocytosis by incorporating intracellular domains such as CD3ζ, FcRγ, and Megf10, with PI3K motifs further enhancing whole-cell engulfment [174,177,178]. Second-generation CAR Ms aimed to boost antigen presentation and T cell activation, using adenoviral vectors to promote stable M1 polarization and employing iPSC-derived CAR Ms for scalable expansion [171]. Third-generation CAR Ms integrate nanotechnology for in vivo reprogramming, leveraging macrophage-targeted nanocarriers and RP-182 peptide-conjugated DNA nanocomplexes to deliver CAR constructs and convert tumor-associated macrophages into a pro-inflammatory, antitumorigenic phenotype [177,179,180].

### 7.2. Functional Advantages and Challenges of CAR Ms

M1-polarized macrophages, in particular, are attractive candidates for CAR engineering due to their intrinsic pro-inflammatory and antitumor properties, including direct tumor cell phagocytosis, antigen presentation to Th1 cells, and cytokine secretion that supports antitumor immunity. Unlike endogenous TAMs which are frequently skewed toward an immunosuppressive M2 phenotype by tumor-derived signals such as CD47, engineered CAR Ms exhibit stable M1 polarization, especially CAR Ms generated using adenoviral vectors which are inherently pro-inflammatory and can stimulate innate immune signaling [166]. Disruption of inhibitory pathways like the CD47–SIRPα axis may further enhance CAR M function within the suppressive TME [181,182,183].

However, several technical and biological challenges must be addressed to realize the full clinical potential of CAR Ms. Limited identification of tumor-specific or tumor-associated antigens (TSAs/TAAs) constrains targeting scope and heightens the risk of on-target, off-tumor toxicity, while antigen escape remains a shared limitation with CAR T therapy [174,184,185]. Additionally, CAR Ms face hurdles such as low transduction efficiency using conventional viral vectors, difficulty sustaining M1 phenotypes in hostile TMEs, and risks like CRS [186,187]. To overcome these barriers, advanced gene delivery methods such as Vpx-lentiviral particles, Ad5f35 vectors, as well as advanced lipid- and polymer-based formulations (such as PEI) have been employed to enhance CAR expression [167,169,188]. Functional enhancements include M2-to-M1 reprogramming, generation of CAR-induced macrophages (CAR-iMacs), and targeted designs expressing chemokines like CCL19 or engaging molecules such as CD147 to improve persistence and TME trafficking. Moreover, synergistic effects have been achieved through combination therapies with monoclonal antibodies (e.g., anti-CD47, anti-CD20, or TAA-specific antibodies) [135].

### 7.3. Recent Advances in CAR Ms’ Antitumor Activity

To date, preclinical studies on CAR Ms have shown promising antitumor activity. Zhang et al. developed iPSCs-derived, CAR-expressing macrophage cells (CAR-iMacs) [171]. Expression of the CAR construct in these iPSC-derived macrophages conferred antigen-dependent effector functions, including cytokine production and secretion, polarization towards a pro-inflammatory/antitumor phenotype, enhanced phagocytic activity against tumor cells, and demonstrable in vivo anti-cancer efficacy. This technological platform provides a scalable source of engineered CAR-iMacs with the potential for cancer cell elimination. Lei et al. developed a novel strategy for enhancing the antitumor efficacy of induced pluripotent stem cell-derived macrophages (iMacs) through genetic engineering [172]. They introduced CARs incorporating a toll-like receptor intracellular Toll/IL-1R (TIR) domain, and this modification resulted in a significant augmentation of antitumor activity compared to first-generation CAR Ms. Collectively, the work by Lei et al. established a second-generation CAR-iMac platform exhibiting enhanced orthogonal phagocytosis and polarization capabilities, leading to superior antitumor functions in the context of solid tumor therapy relative to their first-generation counterparts. In another study, Shah et al., developed human iPSC-derived CAR macrophages targeting prostate stem cell antigen (PSCA) (CAR-iMacs), which express membrane-bound IL-15 and truncated epidermal growth factor receptor (EGFR) for immune cell activation and a suicide switch, respectively [168]. These allogeneic CAR-iMacs exhibit strong antitumor activity against human pancreatic solid tumors in vitro and in vivo, leading to reduced tumor burden and improved survival in a pancreatic cancer mouse model. In a separate study, Liu et al. showed that CAR Ms synergize with CAR T cells in vitro [189]. The synergistic effect could be ascribed to a feedback loop, in which the inflammatory factors secreted by CAR T augment the cytotoxicity of CAR Ms by inducing macrophage M1 polarization and increase the expression of co-stimulatory ligands on CAR Ms, that may promote the fitness and activation of CAR T cells in turn.

Clinical studies of CAR Ms are progressing, and a clinical trial (NCT04660929) has yielded preliminary results: among 14 patients with advanced cancer who did not respond to HER2 monoclonal antibody-targeted therapy, four achieved remission after CAR M treatment without significant CRS or neurotoxicity [190]. Despite limited clinical experience, these collective innovations underscore the growing potential of CAR M therapy as a transformative approach for solid tumor immunotherapy.

## 8. CAR Dendritic Cells (CAR DCs)

CAR DCs represent an emerging frontier in cancer immunotherapy, combining the antigen-presenting power of dendritic cells with the specificity and modularity of CAR technology (Table 2 and Figure 3). Unlike CAR T cells, which directly kill tumor cells, CAR-engineered DCs are designed to enhance adaptive immune responses by presenting tumor antigens to T cells in a highly targeted and immunostimulatory context (Figure 2D). Natural DCs are very rare in peripheral blood, representing <1% of PBMCs. Because of this low abundance, DC-based therapies typically rely on monocyte-derived DCs (moDCs), which are generated ex vivo by culturing CD14^+^ monocytes with GM-CSF and IL-4 [191]. Studies have explored CARs fused to DC-specific signaling domains (e.g., CD40) to enhance T cell priming [192]. Duan et al. developed a novel adoptive dendritic cell, M-DCTNF, which expresses membrane-anchored Muc1 monoclonal Ab, scFv, to target a broad range of breast cancers [10]. M-DCTNF produces TNFα locally to kill cancer cells in combination with the IAP antagonist, SM-164, which degrades IAP proteins. Importantly, unlike CAR T cells, these engineered dendritic cells (M-DCTNF) are not activated to produce a wide variety of cytokines, except for additional overexpressed TNFα, and thus could avoid the severe side effects such as cytokine release syndrome. In another study, Ghasemi et al. established a cell-therapy platform based on mouse or human DC progenitors (DCPs) engineered to produce two immunostimulatory cytokines, IL-12 and FLT3L [170]. Cytokine-armed DCPs differentiated into conventional type-I DCs (cDC1) and suppressed tumor growth in melanoma and liver models. Moreover, cytokine-armed DCPs synergized effectively with anti-GD2 CAR T cells in eradicating intracranial gliomas in mice, illustrating their potential in combination therapies. Ongoing efforts are also aimed at developing iPSC-DCs expressing CAR as an alternative or supporting therapy for solid tumors, although further work is still needed to improve DC differentiation, persistence, improved electroporation-based techniques, and ex vivo transduction efficiencies for the introduction of CAR constructs. Notably, the first-in-human Phase I trial (NCT05631886) is currently underway, evaluating autologous TP53–EphA2 CAR-DCs in combination with immune checkpoint inhibitors in patients with advanced solid tumors or relapsed/refractory lymphomas, marking an important step toward clinical translation of this platform.

## 9. Is There an Ideal Host Cell Type for CAR Cell Therapies in Solid Tumors?

The ideal host cell for CAR cell therapies in solid tumors should balance efficacy, safety, persistence, and adaptability within the hostile TME. While autologous CAR αβ T cells are the most commonly used in current CAR T applications due to their robust cytotoxic function and long-term persistence, they are often prone to exhaustion and have limited tumor infiltration in solid settings [193]. Therefore, alternative immune cell types are being actively explored as potentially superior hosts for treating solid tumors. CAR γδ T cells offer MHC-independent tumor recognition and are less likely to cause GvHD, making them attractive for allogeneic approaches [118]. Memory T cells (T_CM_ and T_SCM_) represent another promising subset due to their long-term in vivo persistence, robust self-renewal capacity, and superior in vivo expansion and antitumor activity, but technical barriers to their isolation and expansion remain [194]. NK cells also exhibit innate tumor cytotoxicity with low GvHD risk and are well-suited for “off-the-shelf” therapies, though they tend to have shorter persistence and are less amenable to genetic modification. CAR Ms provide a promising alternative due to their natural tumor infiltration and capacity to remodel the TME, although they are difficult to expand and manipulate genetically. iPSC-derived immune cells allow scalable, uniform production with precise genetic editing at the pluripotent stage. However, they remain in early development and face manufacturing and maturation challenges. While CAR αβ T cells remain the clinical standard, the ideal host cell for CAR cell therapies in solid tumors may vary depending on therapeutic context. The field is actively developing highly programmable, modular, and controllable CAR systems. Continued research into alternative cell types is essential to optimize safety, efficacy, and scalability in next-generation CAR cell therapies and, more likely, a combination approach would be best-suited depending on the cancer type and patient profile.

## 10. Conditioning Regimens in CAR Cell Therapy

Lymphodepleting conditioning regimens are a critical component of CAR T cell therapy, administered prior to the infusion of engineered immune cells. The primary objective of these regimens is to modify the host immune environment to enhance the engraftment, expansion, and persistence of the infused CAR-modified cells [195]. Typically, conditioning involves the use of chemotherapeutic agents, most commonly a combination of fludarabine and cyclophosphamide (Flu/Cy), both of which contribute to the creation of an immunologically permissive environment [26]. Fludarabine exerts its effects through inhibition of DNA synthesis, leading to lymphocyte depletion and impaired proliferation of host immune cells [196]. Cyclophosphamide, a potent alkylating agent, provides broad-spectrum myeloablation and reduces the risk of host anti-CAR immune responses [197]. Dosing for fludarabine typically ranges from 25 to 150 mg/m^2^, while cyclophosphamide is administered at doses between 300 and 1500 mg/m^2^, with some studies using weight-based dosing, such as 120 mg/kg for pediatric patients [26]. This regimen is designed to deplete endogenous γδ T cells, including regulatory T cells and other competing immune cells, thereby reducing immunologic barriers to CAR cell engraftment. Moreover, lymphodepletion promotes the release of homeostatic cytokines such as interleukin-7 (IL-7) and interleukin-15 (IL-15), which support the survival, proliferation, and functionality of CAR cells post-infusion [198,199].

Although fludarabine and cyclophosphamide are standard for most conditioning protocols, alternative conditioning regimens are under investigation. For example, in trials involving CD52-knockout allogeneic CAR T cells, the anti-CD52 monoclonal antibody alemtuzumab has been incorporated to further eliminate host T and NK cells and reduce the risk of GvHD [200,201,202]. In the UCART19 clinical trial, alemtuzumab was administered in combination with fludarabine and cyclophosphamide, whereas in the ALLO-715 study, varying doses of alemtuzumab (39, 60, or 90 mg over three days) were used to optimize lymphodepletion [200,201]. Bendamustine has been explored as a substitute for cyclophosphamide in some studies [203], and total body irradiation (TBI), although rarely used, remains an option in specific research settings [204]. There is considerable interest in replacing chemotherapy or radiotherapy with non-genotoxic conditioning regimens. A variety of agents, such as antibody radio-conjugates, antibody-drug conjugates, naked antibodies, and CAR T cells (e.g., targeting CD45, CD33, CD117, or CD123) have been developed to effectively deplete hematopoietic lineages in vivo to enable engraftment [205].

Notably, some CAR γδ T cell and CAR NK therapies may employ reduced-intensity or even conditioning-free regimens, leveraging the lower risk of GvHD and shorter lifespan of these innate-like immune cells. For example, trials involving CAR NK cells typically omit alemtuzumab while maintaining robust efficacy and a favorable safety profile [206]. Similarly, CAR Ms generally do not require conditioning chemotherapy, making macrophages attractive candidates for CAR engineering, especially in settings where immune suppression-related complications need to be minimized.

## 11. Safety Considerations Across CAR Cell Platforms

The remarkable clinical success of CAR T cell therapy has been tempered by the occurrence of treatment-related toxicities, which vary depending on the cell source and engineering strategy. Understanding these safety profiles is critical for guiding clinical translation and improving the therapeutic index of next-generation CAR platforms.

Autologous CAR T cells remain the most widely studied and clinically approved products. Their toxicities are well-described and include CRS, driven by supraphysiologic cytokine secretion, and ICANS characterized by encephalopathy, seizures, or cerebral edema [207]. On-target, off-tumor effects such as B-cell aplasia and secondary hypogammaglobulinemia occur with CD19-directed therapies, along with prolonged cytopenias and infection risk [208]. These complications, though manageable with standardized protocols, represent a major determinant of patient eligibility and clinical outcome.

Allogeneic or “off-the-shelf” CAR T cells offer logistical advantages but introduce distinct risks. The most concerning is GvHD due to residual TCR activity, though gene editing strategies, such as TRAC knockout, reduce this likelihood [200,209]. In contrast, HvG rejection remains a challenge, potentially limiting durability of responses [206]. CRS and ICANS may still develop in the allogeneic setting, albeit with variable incidence compared to autologous products.

Emerging strategies that deliver CAR transgenes directly in vivo (e.g., viral vectors, or nanoparticles) hold promise for simplifying manufacturing but raise unique safety considerations [210]. These include the risk of uncontrolled transduction of off-target cell populations, insertional mutagenesis with integrating vectors, and difficulties in regulating dose, expansion kinetics, and persistence once transduction occurs in the patient. Long-term safety data are still limited, underscoring the need for cautious clinical evaluation.

Beyond T cells, CAR-engineered immune cell types each carry lineage-specific risks. CAR NK cells generally exhibit a favorable safety profile with lower rates of CRS and ICANS but can cause transient cytokine-related toxicities and are limited by short in vivo persistence [211]. CAR macrophages (CAR Ms) may induce excessive inflammatory responses, tissue infiltration, or off-target phagocytosis, while CAR dendritic cells could theoretically drive inappropriate immune activation or autoimmunity. The safety of these alternative platforms remains under active investigation and will require rigorous evaluation in ongoing and future clinical trials.

## 12. Conclusions and Perspectives

It is becoming increasingly evident that the best anti-cancer treatments are unique to each patient and continuously adapting to the dynamic changes in disease progression rather than a “one-size-fits-all” treatment. Most adoptive cell therapies are autologous (i.e., from the patient’s own cells); therefore, the major determinants of the final therapeutic product are the clinical and personal unique features of the patient as a donor. Allogeneic “off-the-shelf” CAR T cells, CAR NK cells, CAR Ms, CAR DCs, and other cellular therapies are currently under development.

Looking ahead, we anticipate that no single CAR-engineered immune cell type will fully address the diverse challenges of solid tumors. However, certain platforms appear particularly promising. CAR NK cells combine a favorable safety profile with innate antitumor cytotoxicity and the potential for scalable “off-the-shelf” manufacturing. CAR macrophages are uniquely suited to infiltrate and remodel the TME, potentially enhancing antigen accessibility and T-cell recruitment. In parallel, CAR γδ T cells and VSTs offer alternative recognition modes that may overcome antigen heterogeneity and MHC restriction. Ultimately, we envision that next-generation therapies will likely rely on rational combinations of engineered lymphoid and myeloid cells, potentially supported by pharmacological or synthetic biology approaches to overcome stromal and immunosuppressive barriers.

The use of personalized genetically modified immune cells will greatly expand in the future, and with that, the need for CAR cell manufacturing capacity and timely access to clean room space would need to expand accordingly to avoid a manufacturing bottleneck. Traditionally, academic institutions have been at the forefront of basic and translational research on adoptive cell therapies. However, the manufacturing capacity at academic facilities is inadequate to support larger Phase III trials. This led to the establishment of centralized commercial manufacturing facilities, but this setup typically means high transport costs, complicated transport logistics, and longer “vein-to-vein” times (median 54 days), which inevitably translates to prohibitive costs and inequity in accessing these potentially transformative treatments. A better model would be the establishment of regional Advanced Therapy Manufacturing Product (ATMP) manufacturing facilities, close to the point of care. Point-of-care manufacturing facilities would greatly speed up the process, with shorter “vein-to-vein” transfusion times and less complicated transport and storage logistics, resulting in more accessible treatment options to patients from diverse countries and geographical locations. With this in mind, centralized protocols for GMP manufacturing, storage, and shipping would need to be harmonized across regional manufacturing facilities to ensure good manufacturing practice (GMP), robust quality control, and product safety across sites, from highly trained personnel.

Artificial intelligence (AI), such as machine learning, is increasingly being applied to accelerate the design and clinical translation of CAR-based therapies. AI-driven algorithms can aid in antigen discovery by mining multi-omic datasets to identify tumor-specific targets with minimal off-tumor expression. Structural modeling and predictive platforms allow in silico optimization of CAR design, including scFv-binding domains, hinge length, and intracellular signaling modules, to balance potency and safety. AI can also improve patient selection and response prediction through integration of genomic, transcriptomic, and imaging data, supporting precision immunotherapy. In manufacturing, machine learning can optimize cell culture conditions and quality control by predicting cell phenotype and functional outcomes from high-dimensional datasets. Finally, AI-guided analysis of real-world and clinical trial data may uncover early biomarkers of toxicity, such as CRS or ICANS, enabling proactive risk mitigation. Together, these applications highlight the potential of AI to streamline the development pipeline and enhance the efficacy and safety of CAR cell therapies.

AI may also guide the optimal sequence of administering therapies. Personalized treatments, such as multitarget CARs, bispecific antibodies, neoantigen-based mRNA vaccines, or specific tumor infiltrating lymphocytes (TILs), may need to be custom-designed for each patient and are likely best developed at regional facilities near the hospitals rather than at centralized manufacturing facilities, for ease of access. Finally, to better understand the molecular determinants of resistance, relevant biomarkers can be elucidated simultaneously for the different compartments that are relevant to CAR T cell therapy: (i) the tumor, identified by ctDNA; (ii) the CAR T cell population, identified by cfCAR19; and (iii) other effector T cells of the host, identified by cfTCR, by next-generation sequencing approaches, as described by Mouhssine et al. [212].

Ongoing efforts are aimed at developing strategies to produce engineered CAR cells with enhanced delivery, persistence, and precision. A multi-disciplinary approach is needed to evolve the next-generation precision medicines for patients with solid tumors, and this requires joint efforts from academia and industry to establish safe, innovative, and sustainable standardized protocols for the manufacturing, scale-up, and administration of engineered CAR cell products to patients. Ultimately, collective efforts should focus on developing affordable, accessible, and transformative treatments that will improve outcomes for cancer patients, which, at the same time, would also inform us to accelerate the design of personalized treatment plans for patients with other difficult-to-cure diseases such as neurological disorders, autoimmune diseases, and other rare syndromes.

## Figures and Tables

**Table 1:** Characteristics of lymphoid engineered cell subtypes.

Cell Type	Hallmark Biomarkers	Typical in Vivo Lifespan *	Isolation/Culture and Engineering	Scale-Up and In Vivo Challenges	Key References
αβ T cells (CAR T)	CD3, CD8, TCRαβ; Memory markers (CCR7/CD45RA/RO)	months–years (memory subsets)	leukapheresis → CD3/CD28 activation; IL-2/7/15; viral or non-viral gene transfer	exhaustion and dysfunction in TME; antigen escape; trafficking into solid tumors; time-/cost-intensive autologous manufacture; CRS/ICANS risk	[2]
Memory T cells (T_CM_, T_EM_, T_SCM_)	T_CM_: CD45RO^+^; CCR7^+^ T_EM_: CCR7^−^ T_SCM_: CD95^+^CD45RA^+^	years (especially T_SCM_)	isolation via FACS/MACS using CCR7/CD45 isoforms; IL-7/IL-15 culture favors memory phenotype; CAR/TCR engineering possible	lower exhaustion risk but harder to expand to clinical doses; preserving phenotype during manufacturing	[72,73,74]
Virus-specific T cells (VSTs)	CD3^+^; TCR specific for viral peptides (e.g., CMV pp65, EBV LMP2, adenovirus Hexon)	months–years	expansion from donor or patient PBMCs using viral peptide pools or infected APCs; CAR modification possible for dual specificity	HLA restriction limits allogeneic use; maintaining antiviral specificity post-engineering; donor screening required	[75,76]
Invariant NKT (iNKT)	TCR Vα24-Jα18/Vβ11 (human); CD3, CD161	weeks–months	α-GalCer-loaded APCs or CD1d-based stimulation; CAR-iNKT programs	rarity in blood; expansion yield; persistence in humans	[77]
γδ T cells (e.g., Vγ9Vδ2)	CD3, TCRγδ; NK-like receptors variably	weeks–months	expand with zoledronate/IPP + IL-2/15; CAR-γδ under development	persistence; homing; donor variability; fewer clinical-grade reagents	[78]
NK cells/CAR NK	CD56^+^ CD3^−^; CD16 variably; killer Ig-like receptors	days–weeks (longer with IL-15 support)	sources: peripheral blood, cord blood, iPSC; feeder-based expansions (e.g., K562-41BBL/mbIL-21); mRNA/viral CARs; membrane-bound IL-15	limited persistence; sensitivity to cryostorage; inhibition by TME; GMP feeder systems/logistics	[79,80,81]
iPSC-derived T/NK	as per lineage; pluripotency QA	variable; under study	directed differentiation; gene edits at iPSC stage; clonally defined banks	maturation state; genomic stability; release testing and comparability	[2,82]

* Approximate ranges; influenced by product design, manufacturing methods (e.g., co-stimulation, cytokine support), and host factors.

**Table 2:** Characteristics of myeloid/innate engineered cells.

Cell Type	Hallmark Biomarkers	Typical In Vivo Lifespan	Isolation/Culture and Engineering Methods	Scale-Up and In Vivo Challenges	Key References
Macrophages/CAR Ms	CD14^+^ (monocytes), CD68^+^, HLA-DR; M1/M2 polarization markers	weeks–months (tissue-resident)	monocytes differentiated with M-CSF/GM-CSF; non-integrating viral vectors often used; polarization controlled with cytokines/agonists	prone to re-programming by tumor microenironment (TME); limited proliferation ex vivo; delivery to solid tumors; durability of engineered phenotype	[166,167,168,169]
Dendritic cells (DC vaccines/engineered DCs)	cDC1: CD141/BDCA3 cDC2: CD1c/BDCA1 pDC: CD303; HLA-DR^+^	days	generated from monocytes with GM-CSF + IL-4; matured with cytokine cocktails; antigen loading using defined peptides, mRNA electroporation, or tumor lysates (providing a broad repertoire of tumor-associated antigens (TAAs), including patient-specific neoantigens)	short half-life; migration to lymph nodes; variability in antigen presentation; batch consistency	[10,170]
iPSC-derived myeloid (macrophage/DC)	as per lineage	variable	differentiation from iPSCs; possible genetic engineering at pluripotent stage; “off-the-shelf” cell banks	maturation/function equivalence to primary cells; genomic stability; batch consistency and release criteria	[171,172]

## Data Availability

Not applicable.

## Abbreviations

AAVs	adeno-associated viruses
ATMP	advanced therapy manufacturing product
CAR	chimeric antigen receptor
CAR DC	chimeric antigen receptor dendritic cell
CAR Ms	chimeric antigen receptor macrophages
CAR NK	chimeric antigen receptor natural killer (cell)
CRS	cytokine release syndrome
DCs	dendritic cells
ECM	extracellular matrix
GMP	good manufacturing practice
GvHD	graft-versus-host disease
HCT	hematopoietic cell transplantation
HSCs	hematopoietic stem cells
HvG	host-versus-graft
ICANS	immune effector cell-associated neurotoxicity syndrome
iNKTs	invariant natural killer T cells
iPSCs	induced pluripotent stem cells
LNPs	lipid nanoparticles
MHC	major histocompatibility complex
NK	natural killer (cell)
PBMCs	peripheral blood mononuclear cells
TAAs	tumor-associated antigens
TAMs	tumor-associated macrophages
TBI	total body irradiation
TCM	central memory T cells
TCR	T cell receptor
TGF-β	transforming growth factor-β
TILs	tumor infiltrating lymphocytes
TME	tumor microenvironment
TSAs	tumor-specific antigens
TSCM	stem cell memory T cells
VSTs	virus-specific T cells
ZFNs	zinc-finger nucleases
